# Pulse Oximeter Performance during Rapid Desaturation

**DOI:** 10.3390/s22114236

**Published:** 2022-06-02

**Authors:** Lenka Horakova, Karel Roubik

**Affiliations:** Department of Biomedical Technology, Faculty of Biomedical Engineering, Czech Technical University in Prague, 272 01 Kladno, Czech Republic; roubik@fbmi.cvut.cz

**Keywords:** pulse oximetry, hypoxemia, oxygen saturation, safety limits, avalanche victim, outdoor experiments

## Abstract

The reliability of pulse oximetry is crucial, especially in cases of rapid changes in body oxygenation. In order to evaluate the performance of pulse oximeters during rapidly developing short periods of concurrent hypoxemia and hypercapnia, 13 healthy volunteers underwent 3 breathing phases during outdoor experiments (39 phases in total), monitored simultaneously by five different pulse oximeters. A significant incongruity in values displayed by the tested pulse oximeters was observed, even when the accuracy declared by the manufacturers were considered. In 28.2% of breathing phases, the five used devices did not show any congruent values. The longest uninterrupted congruent period formed 74.4% of total recorded time. Moreover, the congruent periods were rarely observed during the critical desaturation phase of the experiment. The time difference between the moments when the first and the last pulse oximeter showed the typical study endpoint values of *SpO*_2_ 85% and 75% was 32.1 ± 23.6 s and 24.7 ± 19.3 s, respectively. These results suggest that *SpO*_2_ might not be a reliable parameter as a study endpoint, or more importantly as a safety limit in outdoor experiments. In the design of future studies, more parameters and continuous clinical assessment should be included.

## 1. Introduction

Pulse oximetry is a standard monitoring method assessing oxygenation during anesthesia, critical care, and also in out-of-hospital settings. The peripheral saturation of hemoglobin in blood with oxygen (*SpO*_2_) can be assessed using an ear, finger, forehead, or nostril probe. The most commonly applied one is the finger probe where any finger can be used without significant differences in readings [1]. Although this method is challenged by numerous limitations [1], it is successfully used even in outdoor environment for assessment of acclimatization process at high altitude or development of acute mountain sickness [2].

Non-invasive monitoring of blood oxygen saturation is especially important in situations where rapid changes in oxygenation may occur. In clinical practice, this is crucial, for example, in airway management in anesthesia and critical care [3,4,5,6]. Nevertheless, in some sports disciplines, we can also encounter situations accompanied by rapid desaturation, such as during static apnea practiced by breath-hold divers [7].

Another situation when close monitoring of oxygenation is essential is during physiological experiments, for instance, in simulated avalanche snow breathing trials where the subjects usually experience rapidly developing short periods of hypoxia and hypercapnia. Moreover, the levels of oxygen (O_2_) and carbon dioxide (CO_2_) in the organism often serve as study endpoints. The limits are set at different values; for pulse oximetry from *SpO*_2_ 75% [8,9,10] to 85% [11,12], or even 88% [13] and for end-tidal carbon dioxide (*EtCO*_2_) at 8% [10,14], or 60 mmHg [15].

However, the evaluation of these parameters—*SpO*_2_ and *EtCO*_2_—in simulated avalanche experiments is burdensome [16,17,18]. The reliability of the end-tidal CO_2_ monitoring during simulated avalanche experiments, in which high level of gas rebreathing occurs, has already been assessed by Roubik and Filip [16]. They conducted a laboratory experiment using a snow model with a standard gas analyzer in Datex-Ohmeda S/5 (Datex-Ohmeda, Madison, WI, USA) anesthetic monitor and discovered that in as much as 71% of the time for which the volunteers were breathing into this model, the software of the monitor was displaying incorrect values of *EtCO*_2_, compared with the capnographic curve. Similar behavior was observed with the CareScape B650 (GE Healthcare, Helsinki, Finland) monitor.

An apparent discrepancy among different pulse oximeters has been described in a field avalanche experiment [17]. The specificity of these experiments is that the changes in oxygen saturation occur within a couple of seconds, and the dynamic response capability of the device very likely interferes with the embedded software.

To date, an evaluation of the accuracy of pulse oximetry in simulated avalanche breathing trials, similar to the experiment with capnometry [16], has not been performed. Several studies addressed the correlation of *SpO*_2_ with arterial blood oxygen saturation (*SaO*_2_), the probe location, and the method-limiting factors (e.g., hypoxia, poor perfusion, dyshemoglobinemia, hypothermia, skin pigmentation, and hyperbilirubinemia) [1]. Trivedi and his colleagues [19] tested the performance of pulse oximeters during rapid desaturation and resaturation induced by breathing of a gas mixture containing only 10% of oxygen. None of the tested oximeters displayed constantly accurate values throughout the profound hypoxia. However, no study investigated the *SpO*_2_ monitoring response to hypoxia associated with increased work of breathing—a frequent combination of pathological factors seen in volunteers undergoing outdoor studies assessing the gas exchange in avalanche snow.

The aim of this study is to document the behavior of five different pulse oximeters during short-term rapidly developing changes in oxygenation occurring in breathing experiments in simulated avalanche snow.

## 2. Materials and Methods

The data were recorded as a part of a prospective randomized double-blind crossover field study assessing breathing into simulated avalanche snow and into a snow model—perlite [20]. The study was approved by the Institutional Ethical and Review Board of the Faculty of Biomedical Engineering, Czech Technical University (No. A001/018, issued on 22 January 2018) and registered in ClinicalTrials.gov (NCT03413878, last updated: 25 February 2021). All subjects were asked for their written consent for inclusion before they participated in the study.

All recruited volunteers underwent an entrance examination performed by an experienced physician, including assessment of the past medical history, smoking history, physical examination, and spirometry. The exclusion criteria were a Tiffeneau index (FEV1/FVC ratio) less than 0.70, any acute respiratory infection, and a history of a moderate or severe cardiovascular or respiratory disease.

The subjects were continuously monitored throughout the whole experiment. Datex-Ohmeda S/5 (Datex-Ohmeda, Madison, WI, USA) anesthesia monitor [21] served as a primary monitor of physiological and ventilatory parameters, including peripheral saturation of blood with oxygen (*SpO*_2_). Another vital sign monitor, CareScape B650 (GE Healthcare, Helsinki, Finland) [22], provided additional monitoring of *SpO*_2_. Besides those two anesthetic monitors, there were three other monitoring devices in use: Edan M3B (Edan Instruments, Nanshan, Shenzhen, China) [23], Masimo Radical-7 Pulse CO-Oximeter (Masimo, Irvine, CA, USA) [24] and a hand-held pulse oximeter Nonin PalmSAT 2500 (Nonin Medical Inc., Plymouth, MN, USA) [25]. All devices are certified for medical use, have valid periodic safety and technical checks (including validation on a pulse oximeter tester), and are a property of the Faculty of Biomedical Engineering, Czech Technical University in Prague.

Each subject had *SpO*_2_ levels monitored simultaneously by five different finger oxygen saturation probes, placed on right-hand fingers in a standardized manner, presented in Table 1. The position of the finger probe was not randomized. To eliminate possible erroneous readings due to low perfusion or motion artifacts, the volunteer’s hand with all probes was placed into a preheated insulated glove and the participants were instructed to minimize hand and finger movements during the experiments.

The data from all pulse oximeters and monitors were logged and the screens of the monitors were simultaneously filmed to document the *SpO*_2_ values displayed by all oximeters at the same moment. The response times of the individual oximeters were set to minimal possible averaging (in Table 1); this parameter is used in clinical practice to minimize false alarms, but during rapid changes in *SpO*_2_, minimal setting prevents erroneous readings.

Each volunteer underwent three breathing experiments in a random order: ‘S’—breathing into the snow, ‘PD’—breathing into the dry perlite, and ‘PW’—breathing into the wet perlite. Perlite served as a snow model. During each experiment, the study subject was in a prone position, lying on an insulated mat, connected to all sensors of above-mentioned vital sign monitors. At the initiation of the stabilization phase, the subject was connected to the mouthpiece with a nose clip, breathing the ambient air; ventilation parameters with the gas analysis results were recorded. After five minutes, the customized tubing was attached to a cone-shaped container [26] filled with the tested material (snow or perlite) and the main part—the breathing phase—was initiated. Throughout the whole experiment, a clinical assessment of consciousness level of the volunteer was performed by a supervising physician: the physician asked the subject to calculate simple mathematical operations and show the result using their fingers which were not attached to the pulse oximeter probes.

**Figure 1:**
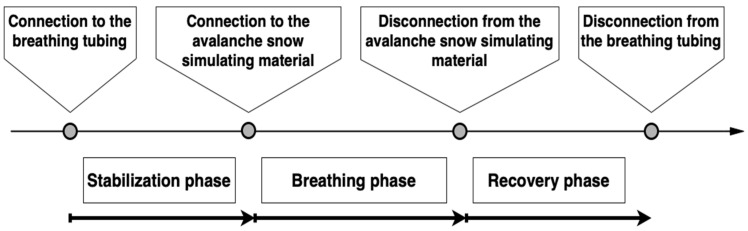
Phases of breathing experiments.

The breathing into the test material was terminated by a subject’s request, by the supervising physician’s command, when the study safety limit was reached (*EtCO*_2_ 62.5 mmHg), or when a gas leak from the tubing was detected using a tracing gas (nitrous oxide). The participant was then disconnected from the test material and allowed to breathe ambient air through the mouthpiece with the respiratory sensor still attached (recovery phase). When all parameters stabilized and returned close to the baseline values, the subject was detached from the mouthpiece and the experiment was ceased. The timeline of the experiment is showed in Figure 1.

The data from pulse oximetry measurements were obtained from simultaneous video recordings of the screens of all the pulse oximeters, in 10-s intervals. All data were processed in MATLAB R2019a (MathWorks, Natick, MA, USA). The values are expressed as mean ± standard deviation, with minimum and maximum values indicated.

Data from all breathing experiments (S, PD, PW) were analyzed together, as according to the already performed analyses [20,26], there are only minor differences in peripheral saturation trends and values among the experiments when subjects breathe into snow and its surrogate materials simulating avalanche snow.

For the analysis, firstly, graphs for all five pulse oximeters measurements in all breathing experiments of all subjects were constructed. Secondly, the graphs were complemented with the interval of accuracy stated by the manufacturers (as summarized in Table 1) [27,28]. Then, the areas of congruent intervals were assessed using an algorithm programmed in MATLAB.

The algorithm for evaluation of the congruency of the *SpO*_2_ signals assessed only those time periods when signals of all five pulse oximeters were present and the accuracy interval was programmed according to manufacturer’s technical specification (in Table 1). In case the measured *SpO*_2_ value was out of the interval for which the manufacturer stated the accuracy, the algorithm used the accuracy stated for the previous interval of peripheral oxygen saturation values.

Finally, all five pulse oximeters were assessed together. Every 60 s, starting at the point when the subject was connected to the breathing circuit (time 0 s), the average value from all *SpO*_2_ measurements from all five pulse oximeters in all subjects was calculated and formed the baseline value. Afterwards, the average for each pulse oximeter for all subjects in all experiments was calculated every 60 s and depicted in the graph with error bars representing standard deviation.

## 3. Results

The clinical trial was conducted between 29 January and 1 February 2018 in Spindleruv Mlyn, Krkonose Mountains, Czech Republic (altitude 762 m above sea level). Written informed consent was obtained from all volunteers before entering the study. All volunteers were members of the Czech Army forces and students at the Military Department of the Faculty of Physical Education and Sport, Charles University in Prague. Thirteen recruited volunteers were fit and well; their characteristics are presented in Table 2.

**Figure 2:**
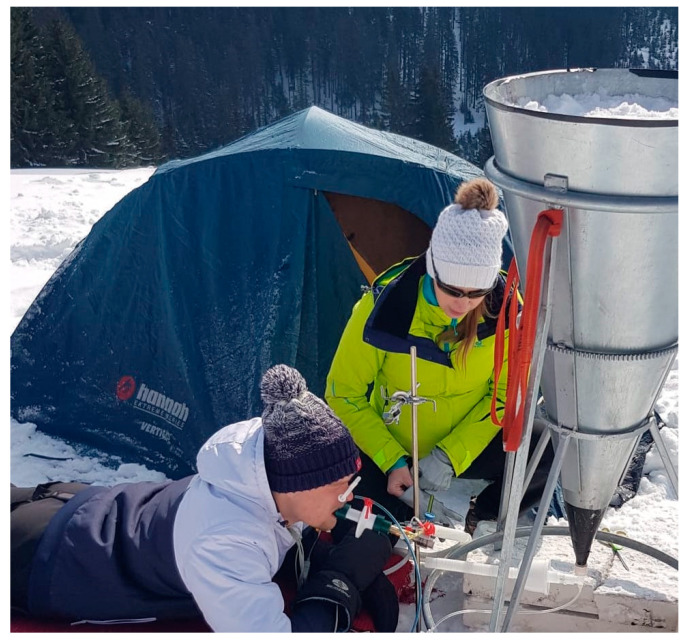
A photo from the stabilization phase of the experiment. Subject is in a prone position, lying on an insulated mat, breathing through a mouthpiece connected to a monitor. The specially designed tubing is ready to be connected to the cone-shaped container filled with material simulating avalanche snow. The subject is monitored closely by the attending physician.

All 13 volunteers were included in the data analysis; in total, 39 breathing experiments were analyzed. The predominant reason for termination of the breathing phase was due to subject’s request (*n* = 24). In five cases, the breathing phase was terminated due to an accidental disconnection of the breathing circuit, due to a detection of the tracing gas—nitrous oxide in the breathing gas—and in the same number of cases the experiment was ceased upon the physician’s decision. No harm occurred to any of the subjects of the experiment. The total length of recorded data in one breathing phase was 419.5 ± 92.4 (230–620) s. A photo from the experiment is in Figure 2.

The oxygen saturation readings displayed by the five different pulse oximeter devices used in this experiment were significantly variable. They varied at the time of onset of desaturation, in the lowest *SpO*_2_ value, and in the duration of the recovery phase, i.e., the period after the subject was disconnected from the test material, breathing ambient air and the oxygen saturation values were returning to baseline. The heart rate and respiratory rate analysis during all phases is available in the Supplementary material.

**Figure 3:**
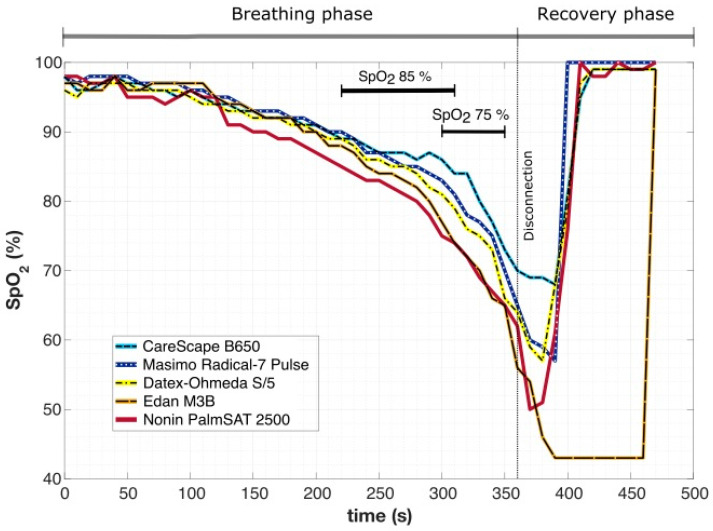
An example of *SpO*_2_ waveforms simultaneously presented by five different pulse oximeters. The time difference between the point when the first and the last pulse oximeter showed the typical study endpoints *SpO*_2_ 85% and 75% is depicted as the black horizontal line. The pulse oximeter Edan M3B showed a stereotypical value of 43% for 70 s after the end of the breathing phase even though other devices presented values within the physiological range already.

An example of changes in *SpO*_2_ over time in one subject during breathing into simulated avalanche snow is presented in Figure 3. The time difference between the moment when the first (Nonin PalmSAT 2500) and the last pulse oximeter (CareScape B650) showed the *SpO*_2_ value of 85% was 90 s. A similar situation occurred at *SpO*_2_ 75%, where the difference was 50 s. The lowest recorded values varied from 69% (CareScape B650) to 43% (Edan M3B), and the screen of Edan M3B was displaying the lowest value constantly for 70 s.

In the whole dataset of all breathing experiments, the time difference between the moment when the first and the last pulse oximeter showed the theoretical study endpoint value of *SpO*_2_ 85% or 75% was 32.1 ± 23.6 s and 24.7 ± 19.3 s, respectively. Moreover, the pulse oximeter embedded in Edan M3B vital sign monitor had a tendency to show the lowest detected *SpO*_2_ value for a prolonged period of time, despite the fact that other four devices were already displaying normal *SpO*_2_ values (as showed in Figure 3). This behavior was observed in 16 out of 39 breathing experiments (in 41% of cases).

When the declared accuracy of the individual pulse oximeter devices was considered (values for each device are in Table 1), in none of the experimental phases did the pulse oximeters show identical values throughout the entirety of the recorded time. Eleven experiments (28.2%) showed no time period when signals from all five pulse oximeters were congruent. Only in one case the devices were in agreement in 86.67% of recorded time. However, on average the congruent periods formed 30.51 ± 26.35 (5.45–86.67) per cent of the recorded time. The total duration of the congruent signals was 115.64 ± 94.00 (30–290) s, with the length of individual segments lasting from 10 s to 260 s. Most often, the signal had two or three separated congruent segments (*n* = 8), seven signals had only one of these segments. The maximum number of observed congruent segments was four in four cases.

**Figure 4:**
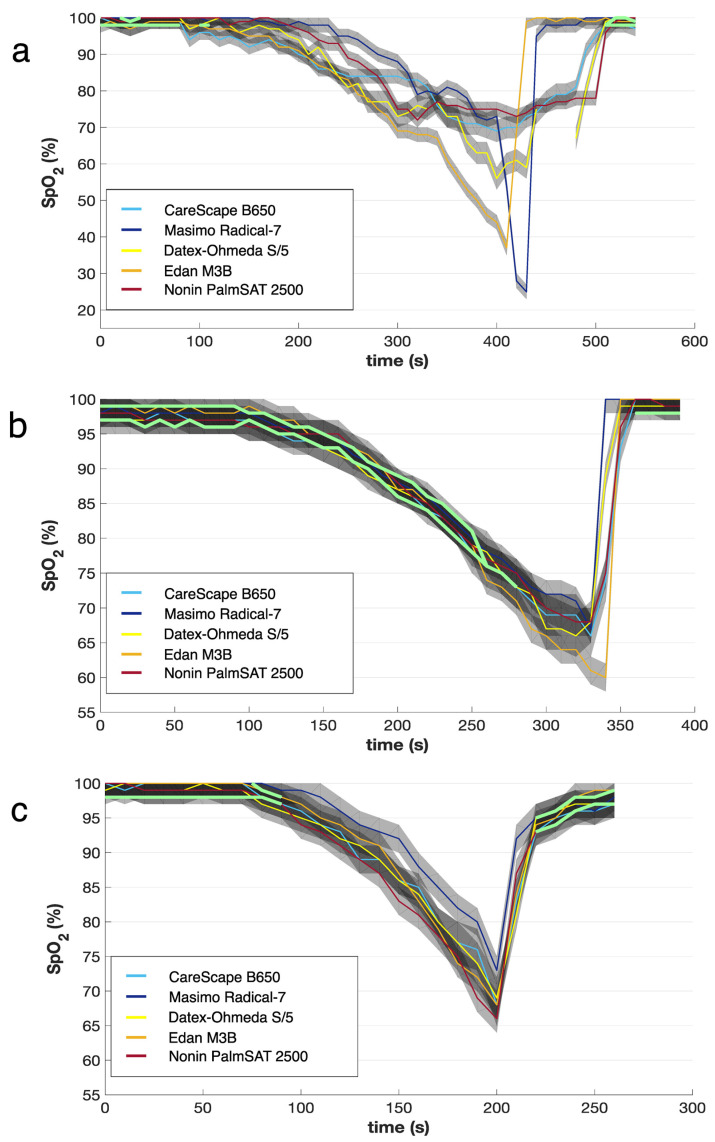
Examples of three individual breathing experiments underwent by three different subjects. The *SpO*_2_ values measured by five different pulse oximeters are presented by color lines. A gray stripe around each line represents the accuracy range of the respective oximeter guaranteed by the manufacturer. Green thick lines represent periods when all five gray stripes overlap, that means all five pulse oximeters showed a value consistent with the others when respecting the accuracies guaranteed by the manufacturer. (**a**) Very short congruent periods; (**b**) long congruent periods lasting 74.4% of experimental time; (**c**) ostensibly long congruent periods were proved to be congruent only in 50% of time; moreover, the congruent segments were present outside the period of rapid changes of *SpO*_2_.

Three examples of evaluation of the congruent segments using an automated algorithm are showed in Figure 4. In Figure 4a the signals are incongruent most of the time, however, there are three short congruent segments (depicted as bright green lines)—two segments at the beginning of the breathing phase and one at the end of the resaturation. The graph in Figure 4b shows the longest uninterrupted congruent segment lasting 260 s with additional 30 s segment at the end of the recovery phase, which forms nearly three quarters of the total recorded time (74.4%). The graph in Figure 4c shows another breathing phase, where the signals seem to be congruent, however, following the analysis, only two congruent segments, lasting in total only 50% of time, were identified. Moreover, these congruent segments were present outside periods of rapid changes in *SpO*_2_.

**Figure 5:**
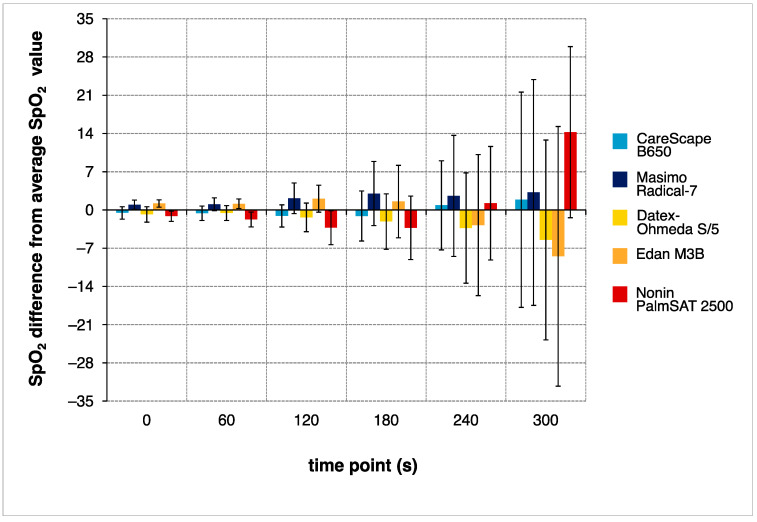
The difference of average *SpO*_2_ value displayed by individual pulse oximetry devices and the average value of all pulse oximeters which represents ‘0’ on the *y*-axis. The difference is displayed at the beginning of the breathing phase (0 s), and at 60, 120, 180, 240 and 300 s. The error bars depict the standard deviation (SD). For example, at 300 s the Nonin PalmSAT on average read 14% higher than the average pulse oximeter reading with a SD 15%.

In Figure 5, all breathing phases were analyzed together and the global difference among the individual pulse oximetry devices is presented. For every 60 s, the difference between the average value displayed by the particular device in all breathing phases and the average value across all the devices is shown. From this graph, it is apparent that with the time course of desaturation, the variance among the devices increases.

## 4. Discussion

The main finding of the study is that oxygen saturation readings displayed by the five pulse oximeter devices during short periods of rapid onset hypoxemia and hypercapnia were significantly different. They varied in the time of desaturation onset, in the lowest measured *SpO*_2_ value, and in the duration of the recovery phase, when the subject was already breathing ambient air and the oxygen saturation was returning to pre-experimental values.

The results suggest that if *SpO*_2_ is chosen as a study endpoint for an outdoor breathing trial, the selection of a particular device can prolong or shorten the trial by tens of seconds (Figure 3). If we consider that most of the volunteers in this study managed to complete 240 s to 300 s of breathing into the material simulating avalanche snow, the change in the testing period by, e.g., 50 s is a significant intrusion into the course of the whole clinical trial.

Not only the rate of the *SpO*_2_ changes, but also the minimal values reached following the disconnection from the test material can pose a significant drawback. Manufacturers usually guarantee the accuracy ± 2% in the interval of *SpO*_2_ 70% to 100% (Masimo Radical-7, Edan M3B, Nonin PalmSAT 2500 [23,24,25]), anesthetic monitors Datex-Ohmeda S/5 and CareScape B650 have declared the accuracy ± 3% in the range between 50% and 80% [21,22] (Table 1). However, even when the declared accuracy of the devices was considered (Figure 4), the values from the pulse oximeters were often not comparable. In fact, in 28.2% of the breathing experiments (*n* = 11) there was no congruent signal identified and in the rest of the experiments, the congruent intervals covered on average only less than a third of the total recorded time (30.51 ± 26.35%). The intervals of congruent signals were observed mainly at the beginning of the breathing phase and at the end during the resaturation. However, in the course of the desaturation, which is the potentially risky experimental phase, the congruity among the devices was infrequent.

The resaturation phase also exhibited considerable differences among the pulse oximeters. Moreover, one device (Edan M3B) had a tendency to show the lowest measured value for a prolonged period of time, whereas the *SpO*_2_ level was within the normal range according to the other devices (as in Figure 3). This behavior can be potentially dangerous because the displayed low value could spur the physician to undertake unnecessary measures. However, the manufacturers guarantee a certain accuracy only to 50% [21,22], or even 70% [23,24,25], and the accuracy of the lower values is questionable.

As a part of the settings of each device, it is possible to select data averaging and display refreshment time, usually referred to as ‘response’. This equates to the speed at which the displayed value appears following the measurement of the parameter. For *SpO*_2_, the monitor can display the values beat-to-beat, or it can present an average of results from the set time period, e.g., 20 s. The latter is a default setting for Datex-Ohmeda S/5 monitors, because in anesthesia it helps to eliminate distracting artifacts and false alarms. However, in breathing experiments, we may observe changes in volunteers’ physiological parameters within a couple of seconds and this averaging can give us incorrect information about the subject’s state and inaccurate experimental data. In addition, this can present safety risks to the volunteers [18]. In this study, the ‘response’ was set to the minimal option available, so it was different for each device (listed also in Table 1): for Datex-Ohmeda S/5 it was set to beat-to-beat, for CareScape B650 to minimum 3 s. In Masimo Radical-7, the values are recorded every 2 s and the response time can be set to minimum 2 to 4 s. The other two devices, Edan M3B and Nonin PalmSAT 2500, do not offer the option of adjustable response time. The difference of the response times among the devices may have affected the simultaneously displayed *SpO*_2_ values [28].

The effect of the *SpO*_2_ averaging time on detection of desaturation events and their duration has already been investigated [29,30]. In the study by McClure and colleagues [30], the change in averaging time from 2 s to 16 s caused significant smoothing of the *SpO*_2_ curves during desaturation periods. The evidence suggests that in experiments with expected rapid changes of *SpO*_2_, devices with minimal response time are preferable to reduce inaccuracy in data acquisition.

Although pulse oximetry is a widely used mean of monitoring, with upgraded algorithms [31], it has well-known limitations [32] and its use outside hospital environment is challenging [2]. The peripheral low perfusion state, typically associated with cold conditions, can alter the pulse oximetry readings. However, during all breathing experiments a maximum effort was made to prevent this effect: the subjects had their hand placed in a warmed glove and the perfusion of the fingers was monitored with use of perfusion index [24]. As there was no significant decrease in the perfusion index observed throughout the breathing experiments [33], the authors speculate that the low perfusion state was not a crucial limiting factor for performance of the pulse oximeters and hence an important source of the incongruity in the displayed *SpO*_2_ values.

Additionally, for a standard in-hospital use, the software is programmed in order to minimize false alarms. This means that rapid brief changes in oxygen saturation are suppressed, as in the hospital settings they are usually caused by motion artifacts, bad connections or poor contact [34,35]. However, these rapid changes in *SpO*_2_ are typical for outdoor breathing experiments in the simulated avalanche snow [8,9,10,11,12,13,14,15] as well as in breath-hold divers [7].

There have been conducted several studies comparing different types of pulse oximeters manufactured by various companies [19,36,37,38,39]. The performance of different pulse oximeters in intensive care setting [36] and in an experiment during severe hypoxemia [19] was compared with arterial blood saturation (*SaO*_2_) as a gold standard. The mean error in *SaO*_2_ measured by pulse oximeters is 3–4% for adults. During hypoxemia with *SaO*_2_ levels below 80% or 90%, the mean error is even more pronounced [40]. It has been hypothesized by Van de Louw et al. [39] that the software algorithm adopted by the particular manufacturer may affect the accuracy of the *SpO*_2_ readings. In addition, for cases of profound hypoxemia, it is challenging to obtain reliable human calibration data [41].

There is contradicting evidence in terms of underreporting or overreporting of *SpO*_2_ by different devices. The tendency of underestimation was found in the interval of *SpO*_2_ 82–93% [36], or even below 75% [19] which was expected by Trivedi and colleagues [19] to be a safety measure, intentionally adopted by the manufacturers. Other studies have revealed an opposite tendency of displaying higher values, for instance due to specifics of the calibration curve used by the software [42]. The difference between *SpO*_2_ and *SaO*_2_ in pediatric data reached the greatest bias in the range of *SpO*_2_ 81–85% (mean 6.6%) [43], exceeding the guaranteed accuracy of the two types of examined devices, in neonates the median size of the bias climbed to 5% in *SpO*_2_ 75–93% [44]. However, to date, there has not been a study examining the bias in adult subjects during outdoor breathing experiments, so the tendency of the pulse oximeters in this scenario is unknown. Moreover, Figure 5 suggests there is no systematic shift in *SpO*_2_ readings in any of the devices, although this analysis may be affected by the performance of one of the pulse oximeters.

During outdoor breathing experiments, the volunteers are standardly monitored by vital sign monitors and additionally they are continuously assessed by an experienced physician. The physician and supervising investigator make decisions regarding the conduction of the experiment based on the physiological parameters presented to them on the screens of vital sign monitors. For this reason, in this study, all analyzed data were obtained from simultaneous video recordings of the pulse oximeters’ screens; purely values that are accessible to the user of the monitor. Raw data recorded directly from the monitors were not used during this analysis.

Although we acquired data for this study during breathing trial with simulated avalanche snow and snow model, our findings are relevant to other clinical situations, where rapid changes in oxygen saturation may occur, e.g., in difficult airway management in anesthesia. In these cases, the physicians also rely on only one physiological parameter, standardly displayed by a single device. Delay in displaying low *SpO*_2_ values may result in a belated appropriate physician’s reaction. On the other hand, repetitive presentation of low values long time after the acute situation ceased—as was exhibited by Edan M3B monitor in 41% of the recorded experiments—can lead to unfitting decisions and improper procedures.

This study examined a specific situation of short rapidly developing periods of desaturation associated with hypercapnia in outdoor environment. The intention was not to analyze the particular pulse oximetry devices and find the most suitable one, but rather to document their behavior during short-term rapid desaturation and resaturation. Additionally, as a standard, the accuracy of pulse oximeters is formally tested during desaturation experiments where subjects experience gradual plateaus of hypoxemia with maximum duration of 10 min [28]—a protocol different from this study. In studies with stepwise protocol, despite periods of desaturation up to *SpO*_2_ of 50% lasting several minutes, the cerebral oxygenation is not critically altered [28,45]. In a recent study by Strapazzon et al. [10], it was shown that whilst breathing into artificial air pockets in avalanche snow, the peripheral pulse oximetry does not correspond to regional cerebral oximetry, measured by near infrared spectrometry. The authors of [43] hypothesize that the cerebral oxygenation may not be impaired despite significantly reduced oxygen supply.

The limitations of our study include mainly the lack of randomization of finger probe placement, or alternatively a simultaneous placement of the same saturation probes on different locations. The pulse oximetry probes were placed on fingers in a standardized manner, the possible differences among fingers could have affected the displayed values, although the variability between fingers is small [1]. An important limitation of this study is also the lack of a gold standard reference for pulse oximeters as *SaO*_2_ repetitively measured in arterial blood samples during a steady state of hypoxemia [28]. However, the nature of this experiment prevents this type of assessment.

Additionally, a restricted number of tested devices and the use of only peripherally placed pulse oximetry probes, which are known to have delayed detection of desaturation compared to centrally placed probes (earlobe, forehead), limited the study. The difference in the response time between the ear probe and the finger probe can be up to 20 s [19]. Another limitation was the different response time of each of the devices, although it was set to the minimal available value. Finally, the number of study subjects was only thirteen, which could be considered a small trial. Although some studies of pulse oximetry accuracy under hypoxic conditions had ten or fewer subjects [19,46,47]. Furthermore, only male subjects were included, even though there is a known difference in *SpO*_2_ values between men and women [48].

Further studies comparing devices currently in use in the clinical practice in hospitals and during outdoor experiments are needed. With the fast development of these monitoring means, testing of the devices in in-hospital and out-of-hospital settings can change the perceived reliability in non-standard situations. Additionally, this study documents that monitoring during short-term changes of peripheral saturation with oxygen has several limitations and clinical assessment by a skillful physician is irreplaceable. Moreover, relying on a single parameter as a study endpoint or a safety limit could not be recommended.

## 5. Conclusions

This study documents that even though standard monitoring equipment is used during outdoor breathing trials, it has notable limitations. The results suggest that *SpO*_2_ might not be a reliable parameter as a study endpoint, or more importantly as a safety limit in outdoor experiments. The irreplaceable role of clinical assessment by a skillful physician should be considered. In the design of future studies, more parameters and continuous clinical assessment should be included.

## Figures and Tables

**Table 1:** A list of used pulse oximetry devices, their standardized placement on subjects’ right-hand fingers, the manufacturer guaranteed accuracy in the defined measurement intervals of peripheral saturation (*SpO_2_*) and the minimal response time set [21,22,23,24,25].

Pulse Oximeter	Finger	Interval of SpO_2_ Measurement	Accuracy in Adults (No Motion)	Response Time (Minimal)
Datex-Ohmeda S/5 anesthesia monitor	V.	40–100%	80–100% ± 2% 50–80% ± 3%	beat-to-beat
Masimo Radical-7 Pulse CO-Oximeter	IV.	0–100%	70–100% ± 2%	3 s
CareScape B650	III.	40–100%	80–100% ± 2% 50–80% ± 3%	2 to 4 s
Edan M3B	II.	0–100%	70–100% ± 2%	not adjustable
Nonin PalmSAT 2500	I.	0–100%	70–100% ± 2%	not adjustable

**Table 2:** The characteristics of the group of volunteers included in the data analysis.

Parameter	Volunteers (*n* = 13)
*Age* (years)	22.8 ± 4.1 (20–35)
*Weight* (kg)	80.8 ± 8.8 (66–103)
*Height* (cm)	179.5 ± 5.0 (172–187)
*BMI* (kg·m^−2^)	25.1 ± 2.6 (22.3–33.3)
*FEV1* (L)	4.6 ± 0.6 (3.3–5.4)
*FVC* (L)	5.0 ± 0.8 (3.3–6.0)
*FEV1/FVC*	0.93 ± 0.05 (0.84–0.96)

The values are presented as mean ± standard deviation and range (minimum–maximum). Abbreviations: *BMI*—Body Mass Index; *FEV1*—Forced Expiratory Volume in 1 s; *FVC*—Forced Vital Capacity.

## Data Availability

The datasets generated and analyzed during the current study are available in the repository at https://ventilation.fbmi.cvut.cz/data/ (accessed on 31 May 2022).

## Supplementary Materials

The following supporting information can be downloaded at: https://www.mdpi.com/article/10.3390/s22114236/s1, Figure S1: The mean heart rate in all subjects during all breathing experiments, error bars show standard deviation. Figure S2: The mean respiratory rate in all subjects during all breathing experiments, error bars show standard deviation. Figure S3: The mean peripheral saturation of blood with oxygen (*SpO_2_*) measured by five different pulse oximeters in all subjects during all breathing experiments, error bars show standard deviation.
