# Supplementary material for: Pulse Oximeter Performance during Rapid Desaturation
Source: Sensors (Basel). 2022 Jun 2;22(11):4236. doi: 10.3390/s22114236 (PMC9185462; doi:10.3390/s22114236)
Supplement: Supplementary file 1 [file sensors-22-04236-s001.zip › sensors-1738952-supplementary.pdf]

Article

Pulse Oximeter Performance during Rapid Desaturation

Supplementary material

Lenka Horakova [1,*] and Karel Roubik [1]

[1] Department of Biomedical Technology, Faculty of Biomedical Engineering, Czech Technical University in Prague, 272 01 Kladno, Czech Republic

* Correspondence: horakle5@fbmi.cvut.cz

Supplementary materials and results

The additional data on heart rate and respiratory rate are presented in Supplementary Figure S1 and S2. The average values of peripheral saturation of hemoglobin in blood with oxygen (*SpO_2*) is presented in Supplementary Figure S3. For all physiological parameters, all subjects and all breathing experiments are analyzed together. Error bars show the standard deviation.

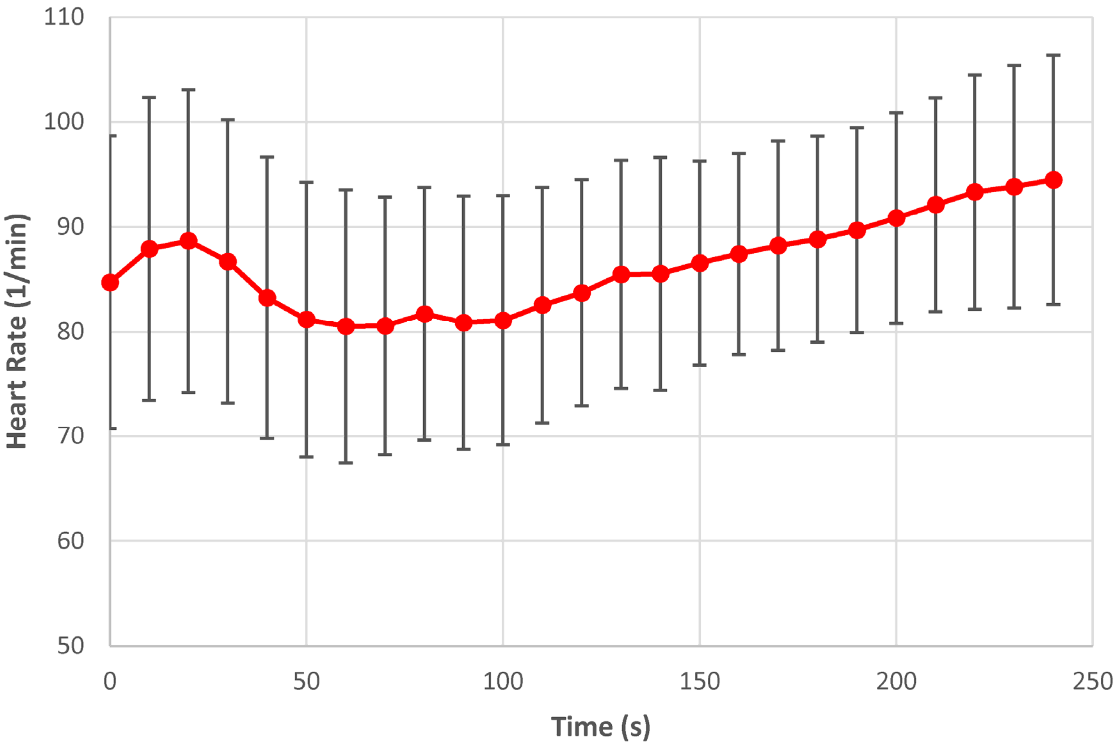

Supplementary Figure S1. The mean heart rate in all subjects during all breathing experiments, error bars show standard deviation.

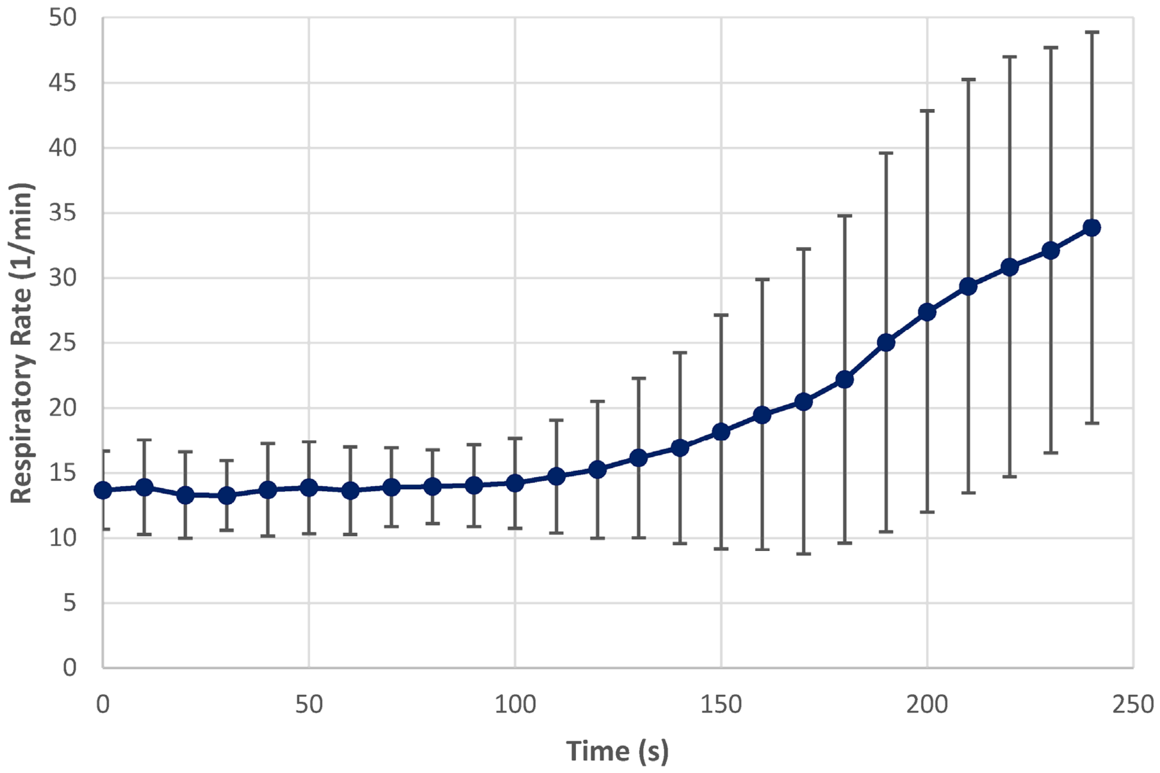

Supplementary Figure S2. The mean respiratory rate in all subjects during all breathing experiments, error bars show standard deviation.

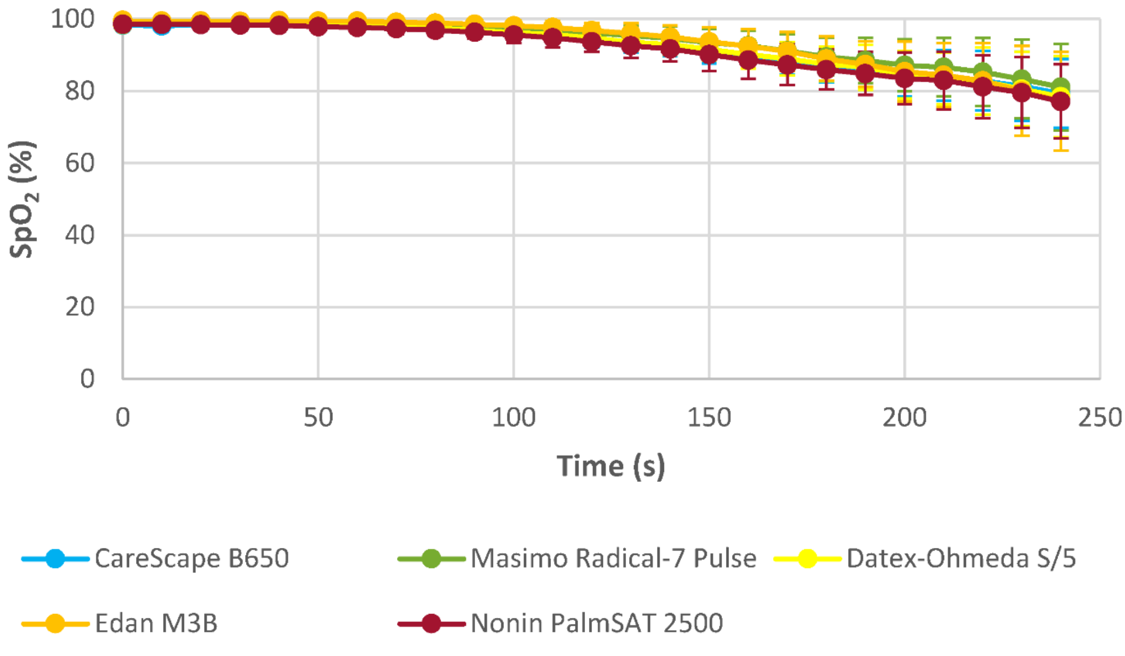

Supplementary Figure S3. The mean peripheral saturation of blood with oxygen (*SpO_2*) measured by five different pulse oximeters in all subjects during all breathing experiments, error bars show standard deviation.
